# Supplementary material for: Enhanced Cognition and Neurogenesis in miR-146b Deficient Mice
Source: Cells. 2022 Jun 22;11(13):2002. doi: 10.3390/cells11132002 (PMC9265316; doi:10.3390/cells11132002)
Supplement: Supplementary file 1 [file cells-11-02002-s001.zip › cells-1776223-supplementary.pdf]

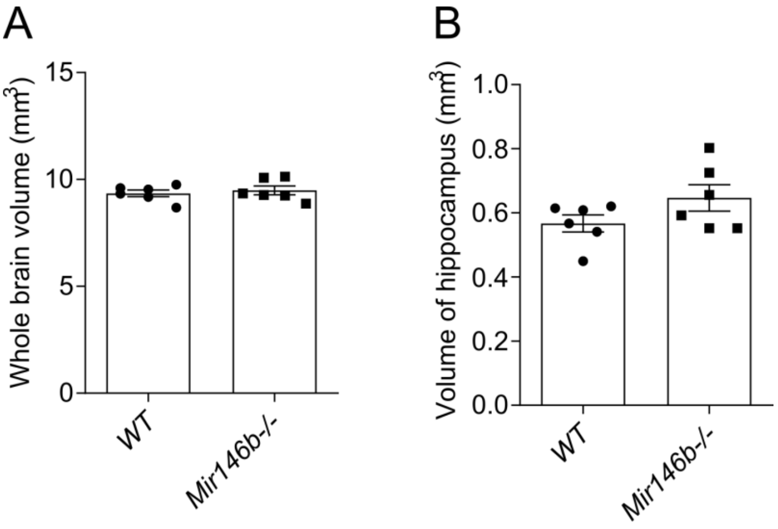

Supplement Figure S1. *Mir146b-/-* mice have no differences in the volume of brain and hippocampus. (**A**) Volume of the whole brain and (**B**) hippocampus of WT and *Mir146b*$^{-/-}$ mice was measured. Data represents as mean ± SEM; Number of animals = 6.

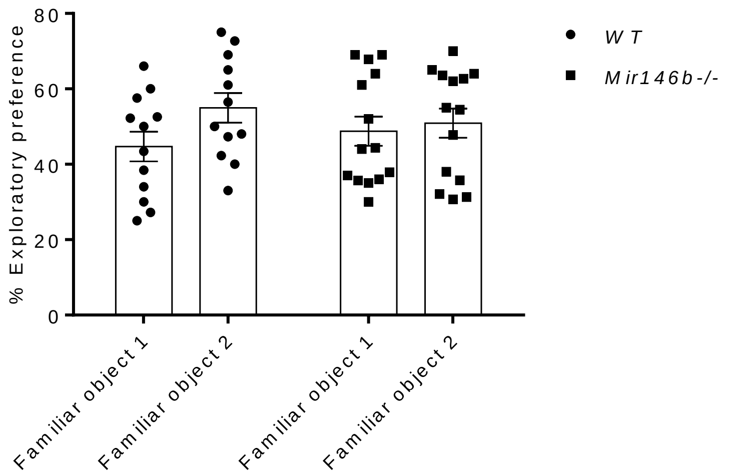

Supplement Figure S2. *Mir146b-/-* and *WT* mice same motivation to explore the objects in NORT. Percentage of exploratory preference was measured in training phase in the presence of two familiar objects. Data represents as mean ± SEM; Number of animals = 11-15

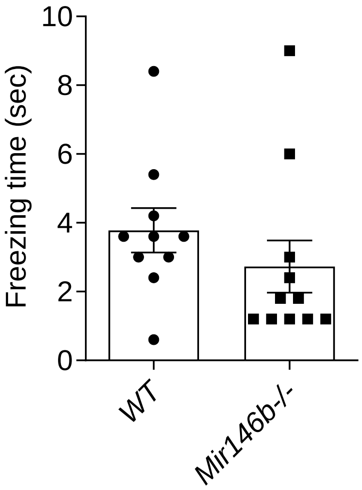

Supplement Figure S3. Baseline freezing of *Mir146b-/-* and *WT* mice in CFC. There were no differences detected in baseline freezing time between *WT* and *Mir146b-/-* mice. Data represents as mean ± SEM; Number of animals = 11-15

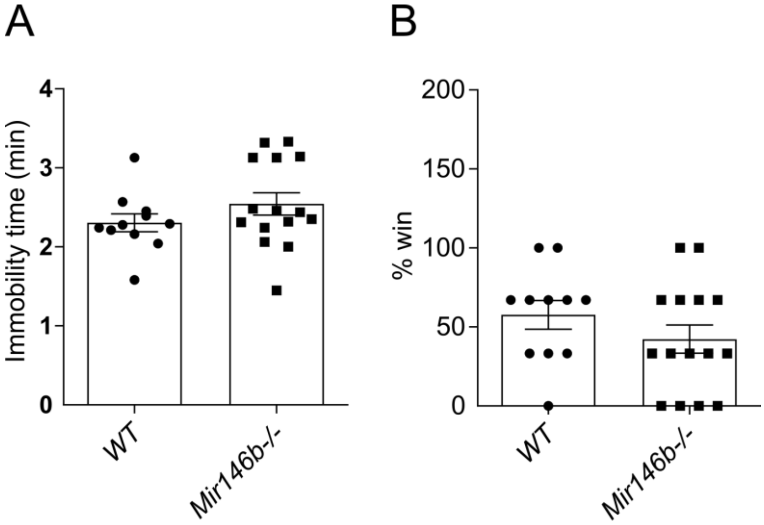

Supplement Figure S4. Mir146b-/- mice have no depression-like behavior and social dominant behavior. (**A**) Immobility time in tail suspension test and (**B**) Percentage win in the tube test of WT and *Mir-146b*$^{-/-}$ mice. Data represents as mean ± SEM; Number of animals = 11-15

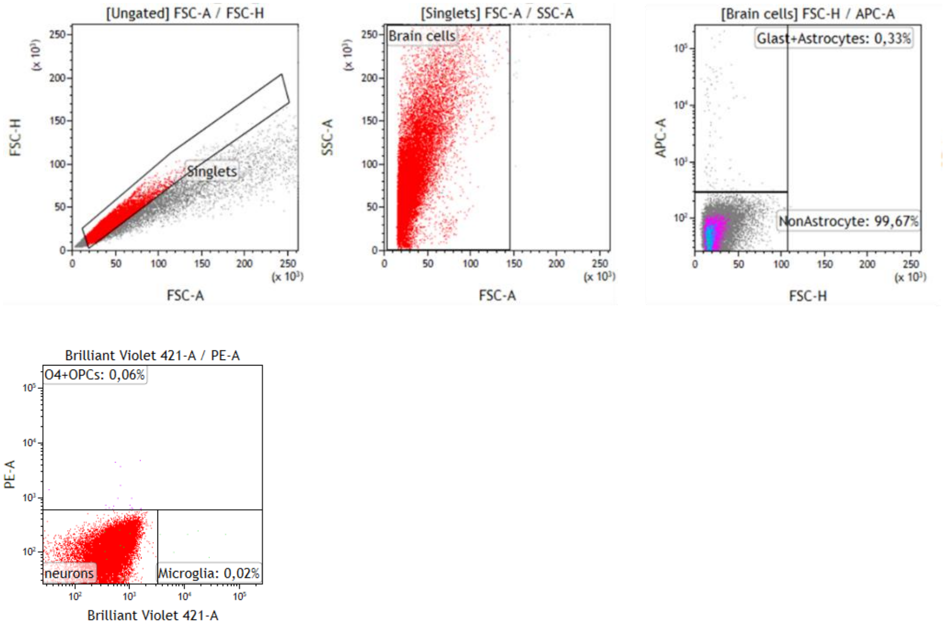

Supplement Figure S5. Representative dot plots of isotype controls used in flow cytometric analysis for gating. GLAST+ astrocytes in APC, O4+ OPCs in PE and CD11b+ microglial cells in BV421 and negative selection for neurons.

Supplementary Table S1. RT-qPCR Primers used in this study

Gene name	Primer Sequence 5′-3′	
CX3CR1	Forward	GAGTATGACGATTCTGCTG
	Reverse	CAGACCGAACGTGAAGACG
SLC17A6 (VGLUT2)	Forward	GCTGGAAAATCCCTCGGACAG
	Reverse	TCGCATAGCGGAGCCTTCTT
GDNF	Forward	CAAAAATCGGGGGTGCGTTT
	Reverse	TCACAGGAACCGCTGCAATA
BDNF	Forward	CATCTGTTGGGGAGACAAGAT
	Reverse	CTTGTCCGTGGACGTTTACTT
IRAK1	Forward	TGTGAGGACACAAGGTGCAA
	Reverse	TAGGCTGGGTGCTTTTCAGG
GAPDH	Forward	GTCATATTTCTCGTGGTTCACACC
	Reverse	CTGAGTATGTCGTGGAGTCTACTG

Supplementary Table S2. Gene ontology groups with neurological function and targeted by miR-146a/b

GO.ID	Description	p-val[A]	Genes
GO: 0022008	neurogenesis	1,494E-05	RARB,NUMB,KLF7,EIF4G2,OSTN,NF2,SH3GL2,**GDNF**,SMAD4,POU3F2,SYT1, BTG2,NRP2,ROBO1,IST1,DGKG,TANC2,PRX,MED1,WASF3,FZD1,MARK1, PHOX2B,SLITRK3,UNC5D,BMPR1A,CAMSAP1,TNIK,FRYL,EDNRB,ERBB4, HEYL,ABL2,LIN28A,MYO6,MAPT,SRRM4,LRP2,RIMS2,SPRY3,STRN,CDON, SEMA3G,TTL,SOX5,PBX2,UBA6,STAU2
GO: 0048699	generation of neurons	3,441E-05	NUMB,KLF7,EIF4G2,OSTN,NF2,SH3GL2,**GDNF**,SMAD4,POU3F2,SYT1,BTG2, NRP2,ROBO1,IST1,DGKG,TANC2,MED1,FZD1,MARK1,PHOX2B,SLITRK3, UNC5D,BMPR1A,CAMSAP1,TNIK,FRYL,EDNRB,ERBB4,HEYL,ABL2,LIN28A, MYO6,MAPT,SRRM4,LRP2,RIMS2,SPRY3,STRN,CDON,SEMA3G,TTL,SOX5, PBX2,UBA6,STAU2
GO: 0030182	neuron differentiatio n	3,796E-05	NUMB,KLF7,EIF4G2,OSTN,SH3GL2,**GDNF**,SMAD4,POU3F2,SYT1, BTG2,NRP2,ROBO1,IST1,DGKG,TANC2,MED1,FZD1,MARK1,PHOX2B,SLITRK 3,UNC5D,CAMSAP1,TNIK,FRYL,EDNRB,ERBB4,HEYL,ABL2,LIN28A,MYO6, MAPT,SRRM4,LRP2,RIMS2,SPRY3,STRN,CDON,SEMA3G,TTL,PBX2,UBA6, STAU2
GO: 0031175	neuron projection development	0,0001063	NUMB,KLF7,EIF4G2,OSTN,SH3GL2,**GDNF**,SMAD4,POU3F2,SYT1,BTG2, NRP2,ROBO1,IST1,DGKG,TANC2,FZD1,MARK1,PHOX2B,SLITRK3,UNC5D, CAMSAP1,TNIK,FRYL,ABL2,MAPT,LRP2,RIMS2,SPRY3,STRN,SEMA3G,TTL, UBA6,STAU2
GO: 0048666	neuron development	0,0001115	NUMB,KLF7,EIF4G2,OSTN,SH3GL2,**GDNF**,SMAD4,POU3F2,SYT1,BTG2,NRP 2,ROBO1,IST1,DGKG,TANC2,FZD1,MARK1,PHOX2B,SLITRK3,UNC5D, CAMSAP1,TNIK,FRYL,EDNRB,ABL2,MAPT,SRRM4,LRP2,RIMS2,SPRY3,STRN, SEMA3G,TTL,PBX2,UBA6,STAU2
GO: 0007399	nervous system development	0,0001884	HNRNPD,TRAF6,RARB,NUMB,KLF7,EIF4G2,ACER3,SCN3B,OSTN,NF2,SH3GL 2,ZNF148,**GDNF**,SMAD4,POU3F2,SYT1,BTG2,NRP2,ROBO1,IST1,DGKG, TANC2,PRX,MED1,WASF3,KDM2B,FZD1,MARK1,PHOX2B,SLITRK3,UNC5D, MYT1,BMPR1A,CAMSAP1,TNIK,FRYL,EDNRB,ERBB4,HEYL,ABL2,LIN28A, QKI,MYO6,MAPT,SRRM4,LRP2,CELF1,CNTFR,RIMS2,SPRY3,STRN,NPAS4, CDON,SEMA3G,TTL,SOX5,PBX2,UBA6,STAU2

[A]The P-value form Fisher exact test showing the significance of the overlap between the target list and indicated functional group.
